# A focus on computer vision for non-contact monitoring of catalyst degradation and product formation kinetics

**DOI:** 10.1039/d3sc90145a

**Published:** 2023-09-27

**Authors:** Niklaas J. Buurma, Scott W. Bagley

**Affiliations:** a Physical Organic Chemistry Centre, School of Chemistry, Cardiff University Main Building, Park Place Cardiff CF10 3AT UK buurma@cardiff.ac.uk; b Pfizer Medicine Design Eastern Point Road Groton CT 06340 USA scott.w.bagley@pfizer.com

## Abstract

Chemists know the value of looking at a reaction for clues about reaction progress and success, but what-it-looks-like has never been quantified. Reid and co-workers (C. Yan, M. Cowie, C. Howcutt, K. M. P. Wheelhouse, N. S. Hodnett, M. Kollie, M. Gildea, M. H. Goodfellow and M. Reid, *Chem. Sci.*, 2023, **14**, 5323–5331, https://doi.org/10.1039/d2sc05702f) have developed an approach that uses camera footage of reactions to obtain quantitative descriptors of changes in reaction mixtures to support kinetic analysis.

## Abstract 2

Cameras keep an eye on your reaction and provide quantitative data to support analysis of reaction kinetics (https://doi.org/10.1039/d2sc05702f).

## Graphical abstract

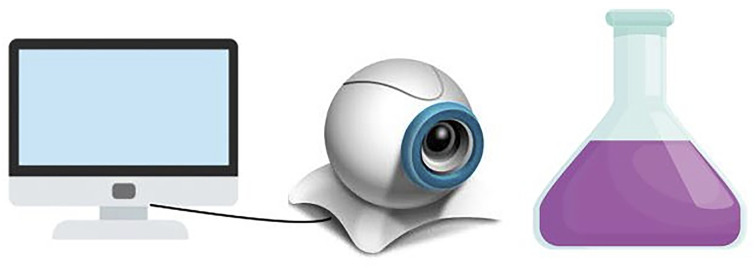

Possibly one of the most-used phrases when discussing chemistry in our teams, in particular when chemistry is proceeding in unexpected ways, is “What does it look like?” Often, this question is followed by a look at a reaction flask, a cuvette, an NMR tube, *etc*. Chemists know there is valuable information in what reactions look like, as noted by Leadbeater *et al.*^1^ “… something that preparative chemists do conventionally is watch their reactions as they proceed”.

“What-it-looks-like data” is popular and for good reason. A variety of chemical reactions and intermediates can be inferred based on colour changes of reaction mixtures. For instance, a deep green or burgundy is commonly seen when Pd(0) ligand complexes are formed, colours from deep yellow to red appear upon metalation or enolate formation; from experience, chemists recognise the bright orange of a successful S_N_Ar producing a nitro arene, the deep blue of a copper coupling, the teal of a rhodium carbene insertion, the catalytic dead-end of palladium black, or the purple of colloidal gold. Beyond such colour changes, another area of visual inspection is of reactant solubility. Has the starting material crashed out? Or the product?

The appearance of reaction mixtures is recorded less frequently than in decades gone by. Instead, an organic chemist now relies on NMR spectroscopy, mass spectrometry, HPLC, and TLC to check reaction progress and to confirm final reaction outcomes. Final isolated products are only summarily described as the almost proverbial off-white solids and colourless oils.

To study reaction progress more rigorously and obtain much-needed kinetic data to support robust mechanistic interpretations,^2^ the aforementioned techniques can be used to generate a reaction profile over time, but moving from reaction progress assessment to kinetic monitoring can be complicated. Kinetic studies require a much higher sampling frequency than checking a reaction to see whether it has finished. Sampling frequency may become problematic in off-line analysis where the higher number of datapoints results in high demand for time on centrally provided equipment, such as NMR equipment, and quenching of reaction mixtures requires additional validation of the analytical method. Where reactions proceed rapidly, on-line analysis becomes the preferred approach. On-line analysis options include NMR spectroscopy, HPLC and UV-visible spectroscopy, but their use is restricted by instrument cost. For example, one may not have exclusive use of an NMR spectrometer to allow on-line reaction monitoring. On-line analysis also requires specialised reaction vessels, such as NMR tubes, HPLC sample vials or quartz cuvettes, and may also require different concentration regimes from synthetic-scale experiments. Consequently, there is often a translation step between kinetic data and what happens in a round-bottom flask or larger reactor.

All in all, we generally fail to accurately record what-it-looks-like data, despite it being a readily available and valuable source of kinetic information. We look at a transition-metal catalysed reaction, where the observable colour spectrum can highlight active oxidation and ligand states. But what do we record? This gap in collection of what-it-looks-like data for kinetic studies has now been addressed by Reid and co-workers (https://doi.org/10.1039/d2sc05702f)^3^ using commercially available cameras coupled with colour analysis.

The use of cameras in chemistry is not new. The increase in popularity of computer vision is driven by commercial cameras and associated data storage becoming cost-effective, while open-source computer vision code OpenCV^4^ with associated online training courses has made computer vision more accessible. The use of computer vision further fits with the trend of increasing use of coding in chemistry and development of custom-made equipment. In their 2013 review on camera-enabled techniques for organic chemistry, Ley *et al.*^5^ distinguished between “camera-assisted viewing”, “computer vision” and “computer vision augmented automation”. As examples of camera-assisted viewing, Leadbeater and co-workers^1^ reported recording reactions in microwave reactors. The reactions included a hydroxide-driven ester hydrolysis (followed through the disappearance of phenolphthalein purple as hydroxide was consumed) and a palladium catalyst decomposing (resulting in spectacular footage of subsequent arcing). The recordings were visually interpreted and no attempt at extracting quantitative data from the videos was reported. Examples of processes that have benefited from forms of computer vision and computer vision augmented automation include automated measurement of gas solubility,^6^ automated filling of reaction vessels,^7^ gravity-based inline liquid–liquid separation,^8^ and automated acid–base titrations.^9^ Beyond these categories, examples include recognition of materials and vessels in a lab^10^ and (stretching the definition of a camera) automated classification of nanoparticles in electron microscopy through computer vision.^11^

The approach developed by Reid *et al.*^3^ differs from previous camera use in chemistry as it represents a significant step towards using cameras to record quantitative data for online and *in situ* reaction monitoring purposes to support physical organic chemistry analyses. The method involves recording a reaction flask using a standard camera and transforming the colours and opacities in the camera footage into a single dimensionless quantitative descriptor of the change in appearance. The selected quantitative descriptor, Δ*E*, is a straight-line displacement in colour space, reflecting a change in colour and contrast irrespective of the actual colours involved. This quantitative descriptor is plotted as a function of time. Although some may argue that this approach is more appropriately categorised as image analysis rather than computer vision, the authors argue that the use of the OpenCV toolbox and subsequent algorithmic analysis moves the work into the area of computer vision for analytical chemistry. In their study, Reid and co-workers interpreted plots of Δ*E* as a function of time to gain insight into catalyst inactivation in a Pd-catalysed reaction. The plots show both gradual and abrupt changes. Temperature and air ingress, as well as the stirring efficiency related to where the reaction vials were placed on a stirrer hotplate, were identified as factors affecting catalyst degradation. Data were cross-validated against data obtained using standard techniques and the authors emphasise that their approach “is built to complement existing analytics, not replace them”. Significantly, this approach does not require sampling from the reaction mixture or specific reaction vessels and therefore reflects how the chemical reaction is carried out in practice. The high temporal resolution of the computer vision data in comparison with off-line sampling means that kinetics details hidden in the bulk are captured. This non-invasive approach uses readily available, low-cost camera equipment, making it scalable, applicable to a variety of settings, and financially accessible to many research teams.

To allow direct quantitative kinetic studies, more work is needed to relate camera observables to concentrations of reactants over time to allow, *e.g.*, fitting of rate equations to the data or to extract reaction orders using Variable Time Normalisation Analysis (VTNA) or Reaction Progress Kinetic Analysis (RPKA).^12^ The required relationship may not be linear, but that doesn't make such a correlation impossible – after all, polymerisation kinetics data can be derived from distinctly non-linear experiments involving viscosity!

This approach offers clear promise to become applicable for reaction monitoring, and likely alternative novel applications, across chemistry. For example, observing the solubility of reactants or initiation of product crystallization will be useful in addition to colour changes indicative of reactive species or undesired pathways. At the same time, even just checking whether a reaction is still being stirred may help explain an unexpected observation. Finally, cameras can go beyond the sensitivity of our eyes. This analysis is therefore not limited to literally human-visible colours. Considering the power of multispectral data analyses deconvoluting UV-visible data and what can be achieved with trained models for computer vision, it is likely that much more data can be extracted in the future. It will be exciting to see what machine-learning approaches can achieve with the rich data recorded by cameras.

It does not take a leap of faith to believe that we are just seeing the beginning of a potential major change, illustrated by the paper by Reid and co-workers. Seeing is no longer believing, seeing is quantitative data.

## Author contributions

Both authors contributed to drafting, writing and finalising this article.

## Conflicts of interest

There are no conflicts to declare.

## Supplementary Material
